# Supplementary material for: Inter-individual differences in the blood pressure lowering effects of dietary nitrate: a randomised double-blind placebo-controlled replicate crossover trial
Source: Eur J Nutr. 2025 Feb 24;64(2):101. doi: 10.1007/s00394-025-03616-x (PMC11850510; doi:10.1007/s00394-025-03616-x)
Supplement: Supplementary file 2 — Supplementary Material 2 [file 394_2025_3616_MOESM2_ESM.docx]

CONSORT extension for reporting N-of-1 trials (CENT) checklist

| Section/Topic | No | Item | Page in  article |
| --- | --- | --- | --- |
| Title and abstract | 1a | Identify as an ‘‘N-of-1 trial’’ in the title  For series; Identify as ‘‘a series of N-of-1 trials’ ’in the title |  |
|  |  |  | 1 N.B. We have used the term ‘replicate crossover’ which most accurately describes the form of n-of-1 trial used |
|  | 1b | For speciﬁc guidance, see CENT guidance for abstracts | 3 |
| Introduction Background and Objectives | 2a.1 | Scientiﬁc background and explanation of rationale | 5-6 |
|  | 2a.2 | Rationale for using N-of-1 approach | 5-6 |
|  | 2b | Speciﬁc objectives or hypotheses | 6 |
| Methods Trial design | 3a | Describe trial design, planned number of periods, and duration of each period(including run-in and wash out, if applicable)In addition for series; Whether and how the design was individualized to each participant, and explain the series design | 7 |
|  | 3b | Important changes to methods after trial start(such as  eligibility criteria),with reasons | N/A |
| Participant(s) | 4a | Diagnosis or disorder, diagnostic criteria comorbid  conditions, and concurrent therapies | 7 |
|  | 4b | Settings and locations where the data were collected. | 7 |
|  | 4c | Whether the trial(s) represents a research study and if  so, whether institutional ethics approval was obtained. | 6-7 |
| Interventions | 5 | The interventions for each period with sufﬁcient details to allow replication, including how and when they were  actually administered | 7-8 |
| Outcomes | 6a.1 | Completely deﬁned pre-speciﬁed primary and secondary outcome measures, including how and when  they were assessed | 10 |
|  | 6a.2 | Description and measurement properties (validity and  reliability) of outcome assessment tools | N/A |
|  | 6b | Any changes to trial outcomes after the trial  commenced, with reasons | N/A |
| Sample size | 7a | How sample size was determined | 10 |
|  | 7b | When applicable, explanation of any interim analyses  and stopping guidelines | N/A |
| Randomization  Sequence generation | 8a | Whether the order of treatment periods was  randomized, with rationale, and method used to generate allocation sequence | 7 |
|  | 8b | When applicable, type of randomization; details of any  restrictions (such as pairs, blocking) | 7 |
|  | 8c | Full, intended sequence of periods. | 7 and supplementary material |

| Allocation concealment mechanism | 9 | Mechanism used to implement the random allocation sequence (such as sequentially numbered containers), describing any steps taken to conceal the sequence  until interventions were assigned | 7 |
| --- | --- | --- | --- |
| Implementation | 10 | Who generated the random allocation sequence, who enrolled participants, and who assigned participants to  interventions | 21 |
| Blinding | 11a | If done, who was blinded after assignment to interventions (for example, participants, care providers,  those assessing outcomes) and how | 8 |
|  | 11b | If relevant, description of the similarity of interventions | 8 |
| Statistical methods | 12a | Methods used to summarize data and compare  interventions for primary and secondary outcomes | 10-13 |
|  | 12b | For series; If done, methods of quantitative synthesis of individual trial data, including subgroup analyses, adjusted analyses, and how heterogeneity between participants was assessed (for speciﬁc guidance on reporting syntheses of multiple trials, please consult the  PRISMA Statement) | 10-13 |
|  | 12c | Statistical methods used to account for carry over  effect, period effects, and intra-subject correlation. | 10-12 |
| Results  Participant ﬂow  (a diagram is strongly recommended) | 13a.1 | Number and sequence of periods completed, and any  changes from original plan with reasons | 7 |
|  | 13a.2 | For series; The number of participants who were enrolled, assigned to interventions, and analysed for the  primary outcome | Supplementary material 1 |
|  | 13b | For each group, losses and exclusions after  randomization, together with reasons | Supplementary material 1 |
| Recruitment | 14a | Dates deﬁning the periods of recruitment and follow-up | 7 |
|  | 14b | Whether any periods were stopped early and/or  whether trial was stopped early, with reason(s) | N/A |
| Baseline data | 15 | A table showing baseline demographic and clinical  characteristics for each group | N/A (provided in text) |
| Numbers analysed  Outcomes and estimation | 16 | For each intervention, number of periods analysed.  In addition for series; If quantitative synthesis was performed, number of trials for which data was synthesized | 10-12 |
|  | 17a.1 | For each primary and secondary outcome, results for  each period; an accompanying ﬁgure displaying the trial data is recommended | 13-15 plus Table 1 |
|  | 17a.2 | For each primary and secondary outcome, the estimated effect size and its precision (such as95% conﬁdence interval). In addition for series; If quantitative synthesis was performed, group estimates | 13-15 |

|  |  | of effect and precision for each primary and secondary  outcome |  |
| --- | --- | --- | --- |
|  | 17b | For binary outcomes, presentation of both absolute and  relative effect sizes is recommended | N/A |
| Ancillary analyses | 18 | Results of any other analyses performed,, including assessment of carryover effects ,period effects, intra-subject correlation  In addition for series; If done, results of subgroup or  sensitivity analyses | 13-15 |
| Harms | 19 | All harms or unintended effects for each intervention.  (for speciﬁc guidance see CONSORT for harms) | N/A |
| Discussion  Limitations | 20 | Trial limitations, addressing sources of potential bias,  imprecision, and, if relevant, multiplicity of analyses | 18-19 |
| Generalisability | 21 | Generalizability (external validity, applicability)of the  trial ﬁndings | 18-19 |
| Interpretation | 22 | Interpretation consistent with results, balancing beneﬁts and harms, and considering other relevant  evidence | 16-20 |
| Other information  Registration | 23 | Registration number and name of trial registry | 6 |
| Protocol | 24 | Where the full trial protocol can be accessed, if  available | 6 |
| Funding | 25 | Sources of funding and other support (such as supply  of drugs), role of funders | 21 |
