# Supplementary material for: Inter-individual differences in the blood pressure lowering effects of dietary nitrate: a randomised double-blind placebo-controlled replicate crossover trial
Source: Eur J Nutr. 2025 Feb 24;64(2):101. doi: 10.1007/s00394-025-03616-x (PMC11850510; doi:10.1007/s00394-025-03616-x)
Supplement: Supplementary file 3 — Supplementary Material 3 [file 394_2025_3616_MOESM3_ESM.docx]

Hayes et al. Inter-individual differences in the blood pressure lowering effects of dietary nitrate: A randomised double-blind placebo-controlled replicate crossover trial.

**Supplementary Table 1.** Mean (SD) systolic and diastolic BP values for replicates 1 and 2 for the nitrate and placebo conditions

|  | Nitrate 1 | | Nitrate 2 | | Placebo 1 | | Placebo 2 | |
| --- | --- | --- | --- | --- | --- | --- | --- | --- |
|  | Pre | Post | Pre | Post | Pre | Post | Pre | Post |
| Systolic BP  (mmHg) | 122 (8) | 115 (10) | 121 (8) | 115 (10) | 122 (9) | 122 (9) | 123 (9) | 124 (11) |
| Diastolic BP  (mmHg) | 72 (8) | 66 (10) | 71 (10) | 67 (8) | 71 (10) | 72 (8) | 73 (9) | 73 (11) |

| **Supplementary Table 2.** Estimated marginal means for blood pressure changes by plasma nitrite concentration | | | | | | | |
| --- | --- | --- | --- | --- | --- | --- | --- |
| **Plasma Nitrite (nM)** | **Systolic blood pressure (mmHg)** | | |  | **Diastolic blood pressure (mmHg)** | | |
|  | **∆** | **95%CI** | |  | **∆** | **95%CI** | |
| 200 | -1 | -3 | 2 |  | -1 | -4 | 2 |
| 300 | -2 | -5 | 0 |  | -2 | -4 | 1 |
| 400 | -4 | -6 | -2 |  | -2 | -5 | 0 |
| 500 | -6 | -8 | -3 |  | -3 | -6 | 0 |
| 600 | -7 | -10 | -4 |  | -4 | -7 | 0 |
| 700 | -9 | -12 | -5 |  | -5 | -9 | 0 |
| 800 | -10 | -14 | -7 |  | -5 | -10 | -1 |
| **∆, denotes expected blood pressure changes for a given plasma nitrite concentration; CI, denotes confidence interval.** | | | | | | | |

**
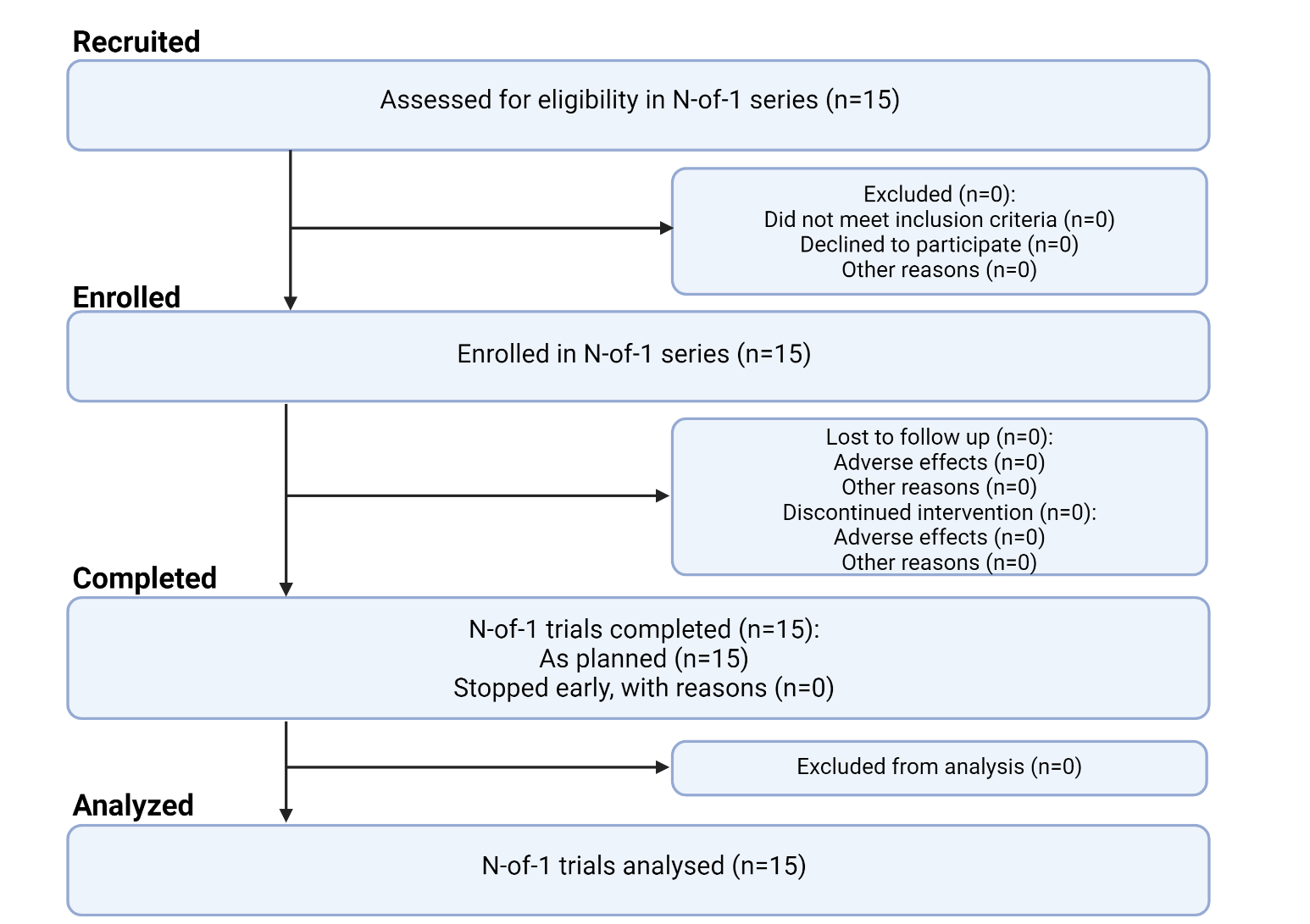
**

**Supplementary Figure 1.** Adapted CONSORT Flow chart according the CENT extension guidelines for n-of-1 trials

**Supplementary Text 1: Randomisation order**

**Source:** Generated using http://www.randomization.com

**Participant 1:** ABBA

**Participant 2:** BBAA

**Participant 3:** ABBA

**Participant 4:** ABAB

**Participant 5:** AABB

**Participant 6:** BAAB

**Participant 7:** BABA

**Participant 8:** BBAA

**Participant 9:** AABB

**Participant 10:** AABB

**Participant 11:** BAAB

**Participant 12:** BBAA

**Participant 13:** BABA

**Participant 14:** BABA

**Participant 15:** BAAB

**Supplementary Text 2: Exploratory statistical analyses**

Additional exploratory analyses involved post-hoc correlations (Pearson’s correlation) estimated to examine relationships between plasma nitrite and nitrate with BP variables. Relationships were further examined using within-participant covariate-adjusted linear mixed effects models including the BP change score as dependent variable, plasma nitrite, condition, period, and the period-by-condition interaction as fixed effects plus a study participant random effect using the *xtmixed* command (StataMP v14.1; StataCorp LP, College Station, TX). Estimated marginal means described the expected BP changes at pre-specified plasma nitrite concentrations whose uncertainty was presented as 95%CI and derived using the *margins* commands [see main manuscript, reference 47].
